# Glut1 Functions in Insulin-Producing Neurons to Regulate Lipid and Carbohydrate Storage in *Drosophila*

**DOI:** 10.3390/biom14081037

**Published:** 2024-08-20

**Authors:** Matthew R. Kauffman, Justin R. DiAngelo

**Affiliations:** Division of Science, Pennsylvania State University, Berks Campus, Reading, PA 19610, USA

**Keywords:** *Drosophila*, Glut1, neurons, Ilp3, Ilp5, TAG, glycogen

## Abstract

Obesity remains one of the largest health problems in the world, arising from the excess storage of triglycerides (TAGs). However, the full complement of genes that are important for regulating TAG storage is not known. The *Glut1* gene encodes a *Drosophila* glucose transporter that has been identified as a potential obesity gene through genetic screening. Yet, the tissue-specific metabolic functions of Glut1 are not fully understood. Here, we characterized the role of Glut1 in the fly brain by decreasing neuronal *Glut1* levels with RNAi and measuring glycogen and TAGs. *Glut1RNAi* flies had decreased TAG and glycogen levels, suggesting a nonautonomous role of Glut1 in the fly brain to regulate nutrient storage. A group of hormones that regulate metabolism and are expressed in the fly brain are *Drosophila* insulin-like peptides (Ilps) 2, 3, and 5. Interestingly, we observed blunted *Ilp3* and *Ilp5* expression in neuronal *Glut1RNAi* flies, suggesting Glut1 functions in insulin-producing neurons (IPCs) to regulate whole-organism TAG and glycogen storage. Consistent with this hypothesis, we also saw fewer TAGs and glycogens and decreased expression of *Ilp3* and *Ilp5* in flies with IPC-specific *Glut1RNAi*. Together, these data suggest Glut1 functions as a nutrient sensor in IPCs, controlling TAG and glycogen storage and regulating systemic energy homeostasis.

## 1. Introduction

Many animal species including mammals have evolved to store excess glucose from the diet as triglycerides (TAGs) in the liver and adipose tissue and as glycogen in muscle and liver cells. While this is a beneficial adaptation in times of food insecurity and unstable environmental conditions, this storage is less useful in developed countries where nutrients are much more readily available. Furthermore, the over-storage of TAGs associated with excess food consumption in these countries has been associated with a variety of metabolic diseases, such as obesity, which continues to prevail as one of the largest health problems in the world [1,2]. Therefore, understanding the genes that are responsible for the storage and metabolism of TAGs could lead to advances in treating these diseases.

The fruit fly, *Drosophila melanogaster*, serves as a model organism for studying both the storage and the metabolism of TAGs due to many of the genes being highly conserved over evolutionary time [3,4]. Still, we know little about the genes responsible for lipid storage. A previous study seeking to identify the genes that are important for lipid storage identified 66 genes that, when mutated, resulted in increased buoyancy and in some cases fat storage [5]. One of these identified genes is *Glut1*, a transmembrane glucose transporter expressed ubiquitously throughout the fruit fly ([5]; FlyBase [6]). Our lab has taken a tissue-specific approach to identifying the metabolic role of Glut1 in the fruit fly, and previous work in our lab has shown that Glut1 in fat cells is important for the storage of TAGs in these cells [7]. However, whether Glut1 plays a role in other metabolic tissues, such as the brain, is not yet known. It is worth noting that, although brain tissue does not include adipocytes, lipid content in brain cells is predominant throughout animal species, being the key component of the axon on which the neural signals rely [8]; therefore, this is of significant functional relevance.

In this study, we sought out to decrease *Glut1* in all *Drosophila* neurons and investigated any impacts on whole-body lipid and glycogen storage. In mammalian species, including humans, Glut1 is typically independent from insulin control, and glucose uptake depends upon insulin-induced cell membrane translocation of mammalian Glut4; meanwhile, in *Drosophila*, which expresses Glut1 and Glut3 but no Glut4, the role of insulin to regulate glucose uptake still needs to be defined. In our study, decreasing *Glut1* pan-neuronally resulted in flies storing less TAG and glycogen than control flies; this was not due to changes in feeding behavior. We also observed a decrease in the expression of the *Drosophila insulin-like peptide* (*Ilp*) *3* and *Ilp5* in the pan-neuronal *Glut1RNAi* flies, suggesting Glut1 plays a role specifically in insulin-producing cells (IPCs). We then targeted *Glut1RNAi* to the IPCs and observed a decrease in TAG and glycogen storage and blunted expression of *Ilp3* and *Ilp5*. Together, these data suggest that Glut1 regulates the uptake of sugar into insulin-producing cells, leading to the expression of *Ilp3* and *Ilp5*, which then regulates TAG and glycogen storage across the whole organism.

## 2. Materials and Methods

### 2.1. Fly Strains and Husbandry

The following fly stocks (and associated Bloomington (BL) or Vienna *Drosophila* Resource Center (VDRC) stock numbers as appropriate) were used in this study: w*; nSyb-Gal4 (BL#51635; [9]), y[1] v[1]; P{y[+t7.7] v[+t1.8] = TRiP.JF01355}attP2 (BL#31603, referred to as UAS-LucRNAi here), y[1] v[1]; P{y[+t7.7] v[+t1.8] = TRiP.JF03060}attP2 (BL#28645, referred to as UAS-Glut1RNAi-TRiP here), w^1118^; UAS-GFP RNAi (BL#9330, referred to as UAS-GFPRNAi here), w^1118^; UAS-Glut1 RNAi (VDRC#13326, referred to as UAS-Glut1RNAi-VDRC here), w*; Ilp2-Gal4 (BL#37516), w[1118] (BL#3605), w[1118]; Tl{w[+mW.hs] = Tl}Ilp3[1] (BL#30882), w[1118]; Tl{w[+mW.hs] = Tl}Ilp5[1] (BL#30884). Flies were grown on standard cornmeal-yeast medium (9 g *Drosophila* agar (Genesee Scientific, San Diego, CA, USA), 100 mL Karo Lite Corn Syrup, 65 g cornmeal, 40 g sucrose, and 25 g whole yeast in 1.25 L water) on a 12 h:12 h light/dark cycle at 25 °C.

### 2.2. TAG, Total Glucose, Free Glucose, and Protein Measurements

Two female flies aged approximately 1 week were homogenized via sonication in lysis buffer (140 mM NaCl; 50 mM Tris-HCl, pH 7.4; 0.1% Triton-X; 1X Pierce Protease Inhibitor EDTA-Free (ThermoFisher, Waltham, MA, USA)). Proteins were measured using the Pierce BCA Protein Assay Kit (ThermoFisher, Waltham, MA, USA), TAGs were measured with the Infinity Triglyceride Reagent (ThermoFisher, Waltham, MA, USA), and total and free glucose were measured with the Pointe-Scientific Glucose Oxidase Reagents (ThermoFisher, Waltham, MA, USA) according to manufacturer’s instructions. Total glucose was assayed by diluting a portion of each sample by half with 8 mg/mL amyloglucosidase (Sigma-Aldrich, St. Louis, MO, USA) in 0.2 M citrate pH 5.0 and incubating for 2 h at 37 °C, while free glucose was measured in samples without amyloglucosidase treatment. Glycogen was calculated by subtracting the free glucose from total glucose. Triglyceride and glycogen values were normalized by protein content.

### 2.3. Café Assay

Feeding was measured as previously described [10]. Briefly, 3 adult female flies were placed in a fly vial with 1% agar and had a 5% sucrose solution in a 5 μL Dummond capillary tube (ThermoFisher, Waltham, MA, USA) as a food source. After 24 h, the amount of solution consumed by the flies was measured and corrected for any evaporation that occurred over the span of the 24 h.

### 2.4. RNA Isolation and qPCR

Total RNA was isolated from approximately 10 female flies using Trizol (ThermoFisher, Waltham, MA, USA) according to the manufacturer’s instructions. A measure of 5 μg of RNA was DNase treated with the DNA-free Kit (ThermoFisher, Waltham, MA, USA) and 0.25 μg of DNase-treated RNA was reverse-transcribed using the qScript Ultra cDNA Supermix (Quanta Biosciences, Beverly, MA, USA) according to the manufacturer’s instructions. The relative expressions of *Ilp2*, *Ilp3*, and *Ilp5* were quantitated using 25 μL reactions including 1 μL cDNA, 2x Perfecta SYBR Green Master Mix (Quanta Biosciences, Beverly, MA, USA), and 300 nM primers under the following conditions: initial denaturation for three minutes at 95 °C, 40 cycles for 30 s at 95 °C, one minute at 60 °C, and 30 s at 72 °C, followed by a melt curve. *Ilp2*, *Ilp3*, and *Ilp5* expressions were normalized by *rp49* expression. Note the following primer sequences: ilp2 F 5′ TCTGCAGTGAAAAGCTCAACGA 3′; ilp2 R 5′ TCGGCACCGGGCATG 3′; ilp3 F 5′ AGAGAACTTTGGACCCCGTGAA 3′; ilp3 R 5′ TGAACCGAACTATCACTCAACAGTCT 3′; ilp5 F 5′ GAGGCACCTTGGGCCTATTC 3′; ilp5 R 5′ CATGTGGTGAGATTCGGAGCTA 3′; rp49 F 5′ GACGCTTCAAGGGACAGTATCTG 3′; rp49 R 5′ AAACGCGGTTCTGCATGAG 3′.

### 2.5. Statistics

For most experiments, averages were compared between control and experimental genotypes using Student’s two-tailed *t*-test using Excel. *Ilp3* and *Ilp5* mutant data were analyzed using a one-way ANOVA with post hoc Tukey test using VassarStats. *p* < 0.05 was considered significant for all statistical tests performed.

## 3. Results

**Figure 1:**
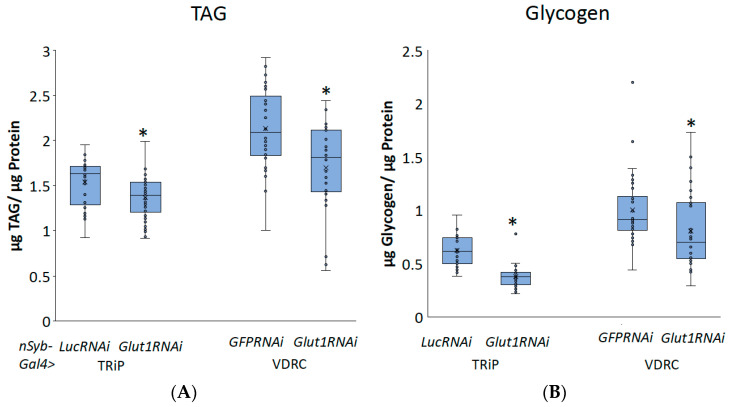
Inducing RNAi to the *Glut1* gene in all neurons in *Drosophila* decreases whole-organism TAG and glycogen storage. (**A**) TAG and (**B**) glycogen normalized by protein content were measured in whole *nSyb-Gal4>Glut1RNAi-TRiP* (*n* = 34) and *nSyb-Gal4>Glut1RNAi-VDRC* (*n* = 30) flies and compared to *nSyb-Gal4>LucRNAi* (*n* = 29) and *nSyb-Gal4>GFPRNAi* (*n* = 30) controls, respectively. Box-and-whisker plots are shown with the x indicating the mean and circles indicating individual data points. Note: * indicates *p* < 0.05, as determined by Student’s *t*-test.

**Figure 2:**
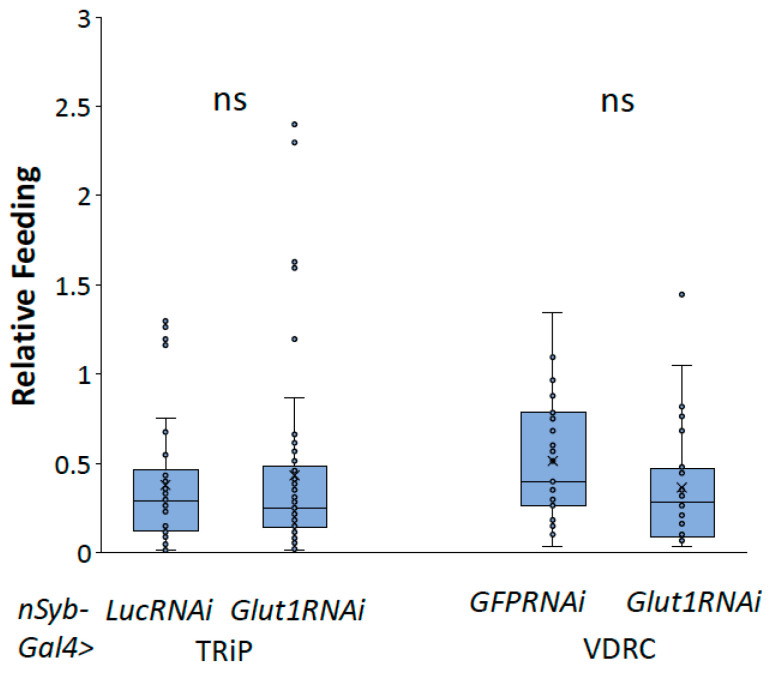
Inducing *Glut1RNAi* in *Drosophila* neurons does not affect food consumption. Relative feeding was measured in *nSyb-Gal4>Glut1RNAi-TRiP* (*n* = 50) and *nSyb-Gal4>Glut1RNAi-VDRC* (*n* = 25) flies and compared to *nSyb-Gal4>LucRNAi* (*n* = 50) and *nSyb-Gal4>GFPRNAi* (*n* = 27) controls, respectively. Box-and-whisker plots are shown with the x indicating the mean and circles indicating individual data points. ns indicates no statistical significance as determined by Student’s *t*-test.

To better understand the role of neuronal Glut1 in *Drosophila* whole-organism TAG and glycogen storage, we decreased *Glut1* in all neurons using *nSyb-Gal4* combined with two different RNAi transgenes targeting *Glut1* and measured TAG or glycogen storage. *nSyb-Gal4* is expressed throughout the *Drosophila* adult nervous system, specifically in the brain and ventral nerve cord [11]. Analysis of triglyceride and glycogen storage of pan-neuronal *Glut1RNAi* revealed a decrease in whole-organism TAG and glycogen storage (Figure 1) that was not due to decreased food consumption (Figure 2). Since similar results were observed with two independent RNAi lines, this further supports the idea that these phenotypes are a result of decreasing *Glut1* expression. Together, these results suggest that Glut1 acts in neurons to regulate organismal nutrient storage.

**Figure 3:**
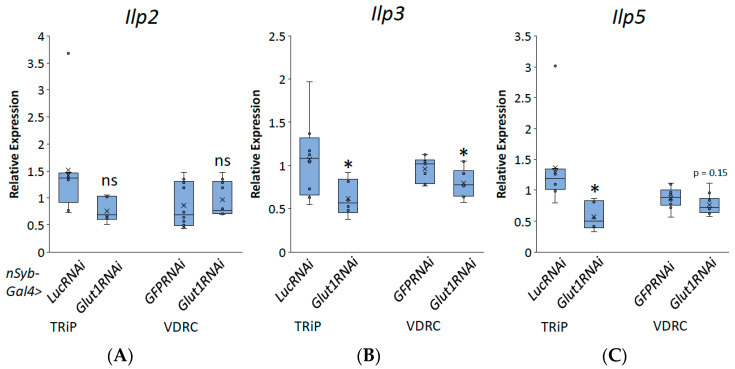
Inducing RNAi to the *Glut1* gene in all *Drosophila* neurons decreases *Ilp3* and *Ilp5* expression. Relative expression of (**A**) *Ilp2*, (**B**) *Ilp3*, and (**C**) *llp5* normalized by relative expression of rp49 was measured in RNA isolated from whole *nSyb-Gal4>Glut1RNAi-TRiP* (*n* = 6) and *nSyb-Gal4>Glut1RNAi-VDRC* (*n* = 10) flies and compared to *nSyb-Gal4>LucRNAi* (*n* = 8) and *nSyb-Gal4>GFPRNAi* (*n* = 10) controls, respectively. Box-and-whisker plots are shown with the x indicating the mean and circles indicating individual data points. Note: * indicates *p* < 0.05 and ns indicates no statistical difference, as determined by Student’s *t*-test.

Decreasing *Glut1* in all neurons specifically led to blunted organismal TAG and glycogen storage; this suggests that Glut1 controls an interaction between the fly brain and the fat tissue. Previous studies have shown that activating the insulin signaling pathway directly in fly fat tissue leads to the accumulation of TAG [12], suggesting that decreasing insulin signaling activity would decrease triglyceride storage. Three *Drosophila* insulin-like peptides (Ilps), *Ilp2*, *Ilp3*, and *Ilp5*, are expressed in the fly brain in insulin-producing cells (IPCs) [13]; when they are secreted, they act on fat tissue to induce TAG and glycogen storage [12,14]. Therefore, we hypothesized that decreasing Glut1 in all neurons altered the expression of *Ilp2*, *Ilp3*, and/or *Ilp5*, which then regulated whole-organism TAG and glycogen storage. To test this hypothesis, we performed qPCR and compared the relative expressions of *Ilp2*, *Ilp3*, and *Ilp5* in pan-neuronal *Glut1RNAi* flies to control flies. *Glut1RNAi* flies had decreased expression of *Ilp3* and potentially *Ilp5*, while *Ilp2* RNA levels were unchanged (Figure 3). These results suggest that Glut1 functions in neurons to regulate *Ilp3* and *Ilp5* expression. It also suggests that Glut1 may have a function specifically in the IPCs to regulate whole-organism nutrient storage.

**Figure 4:**
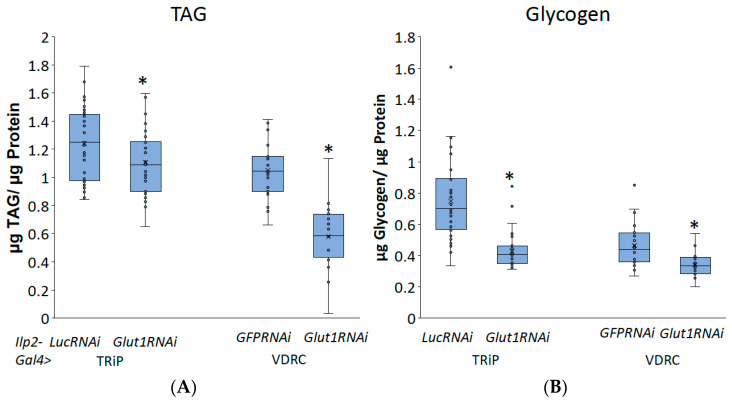
Inducing RNAi in the *Glut1* gene in *Drosophila* insulin-producing neurons decreases whole-organism TAG and glycogen storage. (**A**) TAG and (**B**) glycogen normalized by protein content were measured in whole *Ilp2-Gal4>Glut1RNAi-TRiP* (*n* = 37) and *Ilp2-Gal4>Glut1RNAi-VDRC* (*n* = 24) flies and compared to *Ilp2-Gal4>LucRNAi* (*n* = 33) and *Ilp2-Gal4>GFPRNAi* (*n* = 24) controls, respectively. Box-and-whisker plots are shown with the x indicating the mean and circles indicating individual data points. Note: * indicates *p* < 0.05, as determined by Student’s *t*-test.

**Figure 5:**
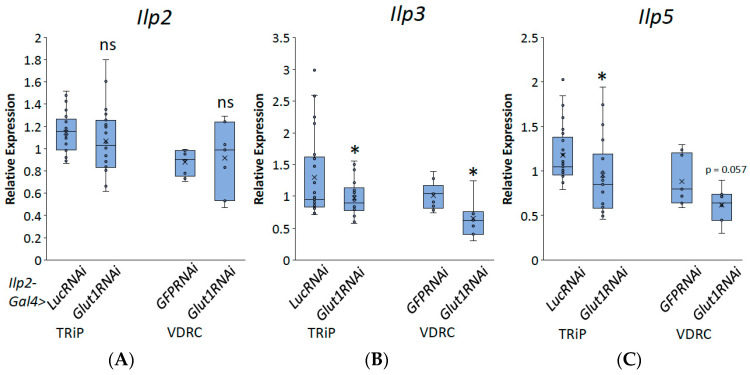
Inducing RNAi in the *Glut1* gene in *Drosophila* insulin-producing neurons decreases *Ilp3* and *Ilp5* expression. Relative expression of (**A**) *Ilp2*, (**B**) *Ilp3*, and (**C**) *Ilp5* normalized by relative expression of rp49 were measured in RNA isolated from whole *Ilp2-Gal4>Glut1RNAi-TRiP* (*n* = 22) and *Ilp2-Gal4>Glut1RNAi-VDRC* (*n* = 7) flies and compared to *Ilp2-Gal4>LucRNAi* (*n* = 25) and *Ilp2-Gal4>GFPRNAi* (*n* = 9) controls, respectively. Box-and-whisker plots are shown with the x indicating the mean. Note: * indicates *p* < 0.05 and ns indicates no statistical significance, as determined by Student’s *t*-test.

To investigate whether Glut1 functions in the IPCs, we induced RNAi to the *Glut1* gene specifically in the IPCs using *Ilp2-Gal4* and measured triglyceride and glycogen storage. Consistent with the pan-neuronal knockdown of *Glut1*, inducing RNAi to the *Glut1* gene specifically in the IPCs resulted in decreased TAG and glycogen storage (Figure 4). This suggests that Glut1 functions specifically in the IPCs to regulate whole-organism TAG and glycogen storage. In addition, decreasing *Glut1* in the IPCs resulted in blunted *Ilp3* and *Ilp5* expression (Figure 5). Together, these results suggest Glut1 functions specifically in IPCs to regulate the uptake of glucose into these cells, which then goes on to regulate the expression of *Ilp3* and *Ilp5*.

**Figure 6:**
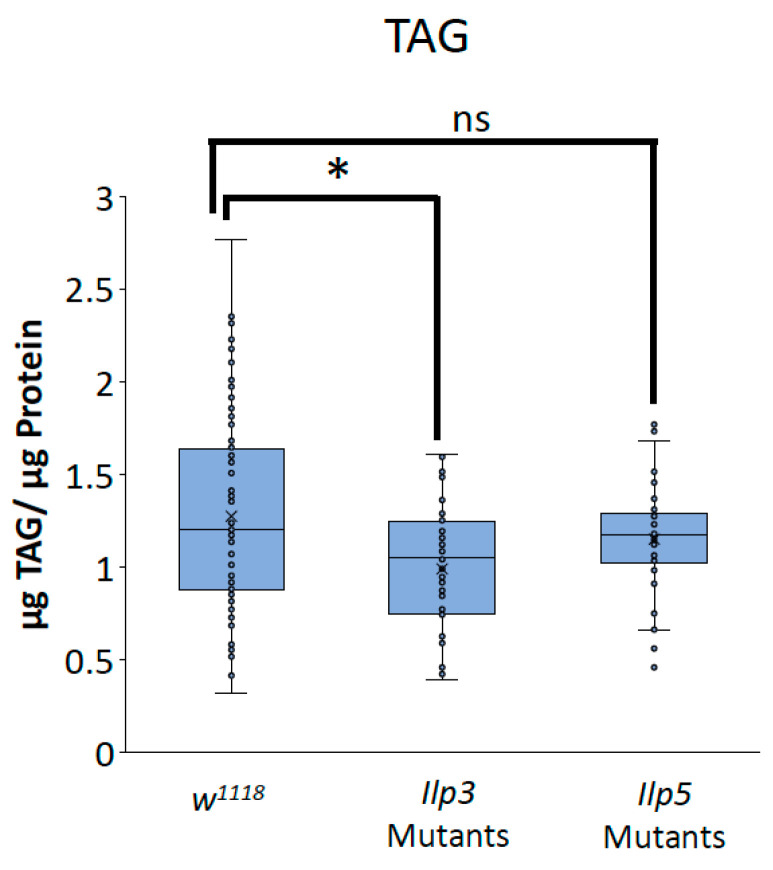
*Ilp3* mutants have blunted TAG storage. TAG normalized by protein content was measured in *Ilp3* mutants (*n* = 48) and *Ilp5* mutants (*n* = 41) and compared to *w^1118^* control flies (*n* = 100). Box-and-whisker plots are shown with the x indicating the mean and circles indicating individual data points. Note: * indicates a *p* < 0.05 and ns indicates no statistical significance, as determined by one-way ANOVA with post hoc Tukey test.

**Figure 7:**
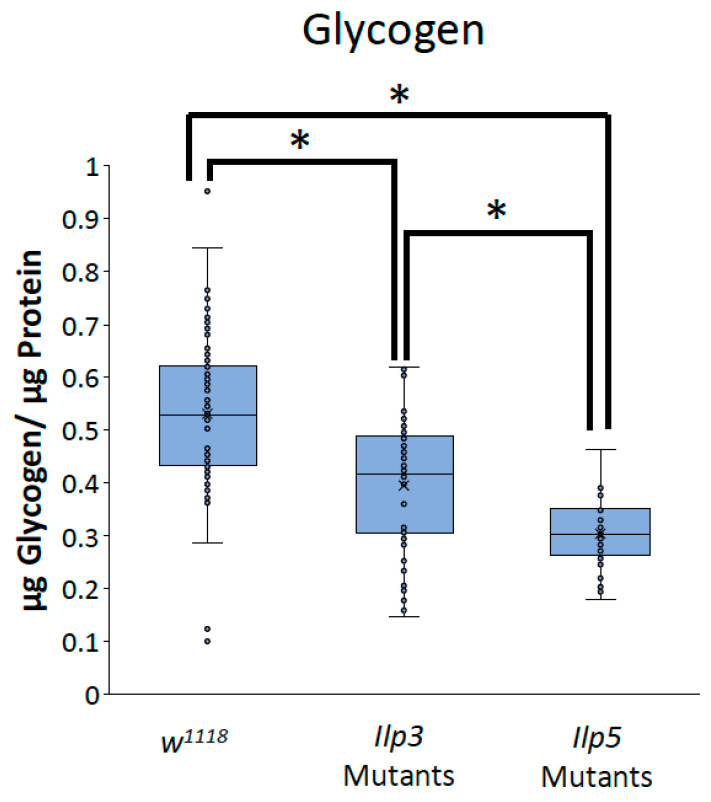
*Ilp3* mutants and *Ilp5* mutants have blunted glycogen storage. Glycogen normalized by protein content was measured in *Ilp3* mutants (*n* = 48) and *Ilp5* mutants (*n* = 41) and compared to *w^1118^* control flies (*n* = 100). Box-and-whisker plots are shown with the x indicating the mean and circles indicating individual data points. Note: * indicates a *p* < 0.05, as determined by one-way ANOVA with post hoc Tukey test.

To determine whether the decreases in TAG and glycogen that were seen when Glut1 was knocked down in the whole brain or IPCs were the result of the reduced *Ilp3* or *Ilp5* expression, we measured TAG and glycogen storage in flies that were mutant for either *Ilp3* or *Ilp5*. *Ilp3* mutants have decreased TAG and glycogen storage compared to control flies, while *Ilp5* mutants have decreased glycogen storage compared to control flies and *Ilp3* mutants (Figure 6 and Figure 7). Together, these results suggest that Glut1 functions in the insulin-producing cells to regulate glucose uptake into these cells, leading to the expression of *Ilp3* and *Ilp5*, which regulates triglyceride and glycogen storage in the whole fruit fly. These data may also suggest that Ilp3 and Ilp5 may differentially affect TAG and glycogen storage.

## 4. Discussion

In this study, the function of neuronal Glut1 and its impact on organismal energy storage was investigated. Here, we show that decreasing *Glut1* in all neurons and specifically in IPCs leads to decreases in organismal TAG and glycogen storage, suggesting that *Glut1* plays an important functional role in the brain to regulate whole-organism nutrient storage in an nonautonomous manner. We also found that decreasing *Glut1* both pan-neuronally and specifically in the IPCs leads to blunted *Ilp3* and *Ilp5* expression. In addition, we show that *Ilp3* mutants have decreased TAG and glycogen, while *Ilp5* mutants have blunted glycogen levels, suggesting that these hormones are important mediators of TAG and glycogen storage. Together, these results suggest that Glut1 functions specifically in IPCs to regulate *Ilp3* and *Ilp5* expression, which are then secreted from these cells to regulate whole-organism nutrient storage.

Previous work from our lab has characterized the role of Glut1 in *Drosophila* adipose tissue. When *Glut1* is decreased specifically in these cells, TAG content decreases and the expression of fatty acid synthase is blunted [7]. These data suggest that, in adipose tissue, Glut1 functions to regulate the synthesis of TAG and fatty acids autonomously [7]. However, the mechanism through which neuronal Glut1 regulates organismal nutrient storage is likely very different. Most TAG storage occurs in the fat of the body [4], so the decreases in nutrient storage observed in this study during a pan-neuronal and IPC-specific *Glut1* knockdown are hypothesized to be happening in the fat of the body nonautonomously. However, previous studies have shown that the induction of the insulin signaling pathway in the fat of the body leads to the accumulation of TAGs [12]. Here, we show that decreases in *Ilp3* and *Ilp5* expressions likely lead to decreases in TAG and glycogen storage; we hypothesize that this decrease in nutrient storage is the result of decreased insulin signaling activity in fat tissue.

Interestingly, *Ilp3* and *Ilp5* mutants were shown to differentially affect nutrient storage; *Ilp3* mutants showed decreases in TAG and glycogen storage, while *Ilp5* mutants only showed decreases in glycogen storage. These data could suggest that *Ilp3* and *Ilp5* differentially signal to affect nutrient storage via a phenomenon known as ligand bias. Ligand bias, also referred to as biased agonism or ligand functional selectivity, allows for ligands to selectively activate a specific response associated with a receptor [15]. While this phenomenon has been predominately studied in G-protein-coupled receptors, recent studies have begun to reveal that receptor tyrosine kinases (RTKs) can also exhibit ligand bias [16,17]. Since fruit flies have multiple Ilps and only one insulin receptor (Drosophila insulin receptor or DInR [18]), it is possible that these Ilps differentially activate DInR to signal through different metabolic pathways to lead to different outputs. While both Ilps are shown to decrease glycogen storage, Ilp3 could bias the resulting signaling pathway towards TAG storage. Furthermore, *Ilp5* mutants have less glycogen storage compared to *Ilp3* mutants. This could suggest that Ilp5 biases the signaling pathway towards glycogen storage more than Ilp3 does. Additional experimentation will be necessary to address these possibilities.

The genetic screen in *Drosophila* larvae that identified the *Glut1* gene as a potential anti-obesity gene reported that a whole-body mutant results in increased buoyancy, suggesting increased TAG storage [5]. These data contradict what is shown here, as inducing RNAi to the *Glut1* gene specifically in neurons leads to decreases in both TAG and glycogen storage. Given that *Glut1* is ubiquitously expressed, it is likely that it functions differently across cell types, and that decreasing *Glut1* in all cell types may alter whole-organism TAG and glycogen storage differently than decreasing it specifically in neurons. Recent research has also shown the sexual dimorphic phenotype observed in adult flies is reversed in larvae; in adult flies, adult females store more TAG than males, while male larvae store more TAG than females [19]. It is therefore possible that the observed differences in TAG storage phenotypes between larva and adult flies when *Glut1* is decreased are due to the underlying differences in TAG metabolism between these stages of development. It is also possible that Glut1 may function differently in larvae than in adults. Furthermore, the previous work from our lab showing a similar decreased TAG and glycogen phenotype when *Glut1* is decreased in adipose tissue supports the idea that Glut1 functions in specific tissues differently than when it is decreased in multiple tissues at once. Experiments designed to address these questions will help clarify the functions of Glut1 throughout the entire *Drosophila* lifecycle.

The decrease in *Ilp3* and *Ilp5* expression seen in the *Glut1RNAi* flies is consistent with previous work investigating the effects of starvation on *Ilp* expression; when larvae are starved, *Ilp3* and *Ilp5* expression is suppressed [20]. Here, we showed that decreasing *Glut1* in neurons leads to decreased *Ilp3* and *Ilp5* expression, potentially due to mimicking the effects of starvation seen in previous studies. This suggests that Glut1 could be serving as a glucose transporter, allowing glucose to enter the IPCs and regulate the function of those cells; this is consistent with previous studies, showing that overexpressing *Glut1* in the fly increases glucose uptake [21]. However, how Glut1 regulates the expression of *Ilp3* and *Ilp5* is not yet known. With the decreases in *Ilp3* and *Ilp5* mRNA levels in *Glut1RNAi* flies, we hypothesize that the activity of a sugar-responsive transcription factor is being regulated via Glut1, but the exact transcription factor is not yet known. One potential sugar responsive transcription factor is *Mondo*, the *Drosophila* homolog of a carbohydrate-response-element-binding protein [22,23]. However, previous studies have shown that decreasing *Mondo* in neurons leads to increases in *Ilp3* mRNA and no changes in *Ilp5* mRNA [11], suggesting that *Mondo* may not be the transcription factor regulating *Ilp* expression in our model. Alternatively, *Drosophila* forkhead box, subgroup O (*dFOXO*) transcription factors could also be regulating the expression of *Ilp3* and *Ilp5* based on nutrient availability. *dFOXO* has been shown to activate during starvation to enhance the transcription of genes that are important for low-nutrient environments, leading to survival [24]. It is possible that decreasing glucose in the brain mimics a starvation state; therefore, it activates *dFOXO* in a similar fashion. This activation of *dFOXO* could then be acting as a transcriptional inhibitor of *Ilp3* and *Ilp5*, leading to the decreases in expression shown here. Further investigation is needed to identify the transcription factor that is being affected by Glut1 in IPCs to regulate *Ilp3* and *Ilp5* expression.

It is also possible that Glut1 is regulating Ilp3 and Ilp5 post-transcriptionally. While we show that there is a decrease in the levels of expression of *Ilp3* and *Ilp5* mRNAs when *Glut1* is decreased in the brain, we do not know whether *Glut1RNAi* is leading to a decrease in the amount of these proteins being created. In addition to fewer *Ilp3* and *Ilp5* mRNAs being made, the level of translation could also be altered in the *Glut1RNAi* flies. The presence of glucose in the liver has been shown to stabilize preproinsulin mRNAs in mammals [25,26]. Therefore, it is possible that glucose entry into *Drosophila* neurons leads to the stabilizing of *Ilp3* and *Ilp5* mRNAs, promoting the translation of Ilp3 and Ilp5 in the IPCs. Furthermore, studies have shown that preproinsulin levels are predominantly regulated by glucose at the translational level and are not dependent on mRNA levels in the pancreatic beta cells of mice [27,28]. Therefore, it is possible that Glut1 may regulate the entrance of glucose into the IPCs to regulate the translation of Ilp3 and Ilp5 via a similar mechanism.

Blunted Ilp secretion in the *Glut1RNAi* flies is another possible cause for the decreased TAG and glycogen storage seen in these flies. A previous study has shown that inducing *Glut1RNAi* specifically to the IPCs leads to decreases in Ilp2 secretion but no changes in total Ilp2 protein level [29]; this finding is consistent with the expression pattern we show here. Thus, decreasing *Glut1* could lead to blunted Ilp3 and Ilp5 secretion as well as Ilp2 secretion. Furthermore, a previous study has shown that the IPCs are excited in the presence of glucose [30]. It is possible that decreasing *Glut1* in the IPCs inhibits the ability of these neurons to fire and secrete Ilps due to decreases in glucose in these cells. Further study is needed to better understand whether the secretion of Ilp3 and Ilp5 is affected in *Glut1RNAi* flies.

IPCs are not the only sugar-responsive neurons present in the fly brain; there are a variety of other neuropeptides that have established roles in regulating metabolism or IPC functions [31]. This suggests that there may be other neuronal populations in which Glut1 may play an important regulatory role. Neuropeptide F, corazonin, and short neuropeptide F have been shown to interact with each other to impact both nutrient storage and to act on brain IPCs [32,33,34,35]. Therefore, it is possible that Glut1 functions in these cells to sense nutrient availability to affect IPC and/or Ilp activity. Leukokinin (Lk) has also been shown to prevent sleep under starved conditions and acts on IPCs, connecting behavior to metabolism [36]. It is possible that Glut1 functions in these cells to detect nutrient availability to affect metabolic regulation of sleep. In addition, adipokinetic hormone (AKH) has various functional similarities to mammalian glucagon, suggesting an important role in nutrient sensing in times of starvation [37,38]. Glut1 may regulate the uptake of glucose into these cells to signal times when the organism is fed or starved to regulate the breakdown of stored TAG. Further study is needed to understand the role of Glut1 in these different neuronal populations.

## 5. Conclusions

In summary, we have shown that Glut1 functions in the *Drosophila* brain to regulate whole-organism nutrient storage. Glut1 regulates the uptake of sugar into insulin-producing cells, leading to the expression of *Ilp3* and *Ilp5*, which then regulate TAG and glycogen storage across the whole organism. Our increased understanding of the neuronal functions of Glut1 helps to broaden our knowledge of how nutrients are detected by the brain and then how the brain signals their storage throughout the whole organism. Understanding the tissue-specific mechanisms by which nutrients are stored will allow us to better understand and combat metabolic diseases, such as obesity.

## Data Availability

Data are contained within the article.
